# Evaluation of the Predictors for Unfavorable Clinical Outcomes of Degenerative Lumbar Spondylolisthesis After Lumbar Interbody Fusion Using Machine Learning

**DOI:** 10.3389/fpubh.2022.835938

**Published:** 2022-03-03

**Authors:** Shengtao Dong, Yinghui Zhu, Hua Yang, Ningyu Tang, Guangyi Huang, Jie Li, Kang Tian

**Affiliations:** ^1^Department of Bone and Joint, First Affiliated Hospital, Dalian Medical University, Dalian, China; ^2^Department of Spine Surgery, Second Affiliated Hospital of Dalian Medical University, Dalian, China; ^3^Department of Orthopedics, Dalian No. 3 People's Hospital, Dalian, China; ^4^Department of Otolaryngology, Head and Neck Surgery, Second Affiliated Hospital of Dalian Medical University, Dalian, China

**Keywords:** degenerative lumbar spondylolisthesis, clinical outcome, machine learning, predictor, lumbar interbody fusion

## Abstract

**Background:**

An increasing number of geriatric patients are suffering from degenerative lumbar spondylolisthesis (DLS) and need a lumbar interbody fusion (LIF) operation to alleviate the symptoms. Our study was performed aiming to determine the predictors that contributed to unfavorable clinical efficacy among patients with DLS after LIF according to the support vector machine (SVM) algorithm.

**Methods:**

A total of 157 patients with single-segment DLS were recruited and performed LIF in our hospital from January 1, 2015 to October 1, 2020. Postoperative functional evaluation, including ODI and VAS were, performed, and endpoint events were defined as significant relief of symptom in the short term (2 weeks postoperatively) and long term (1 year postoperatively). General patient information and radiological data were selected and analyzed for statistical relationships with the endpoint events. The SVM method was used to establish the predictive model.

**Results:**

Among the 157 consecutive patients, a postoperative unfavorable clinical outcome was reported in 26 patients (16.6%) for a short-term cohort and nine patients (5.7%) for a long-term cohort. Based on univariate and multivariate regression analysis, increased disc height (DH), enlarged facet angle (FA), and raised lateral listhesis (LLS) grade were confirmed as the risk factors that hindered patients' short-term functional recovery. Furthermore, long-term functional recovery was significantly associated with DH alone. In combination with the SVM method, a prediction model with consistent and superior predictive performance was achieved with average and maximum areas under the receiver operating characteristic curve (AUC) of 0.88 and 0.96 in the short-term cohort, and 0.78 and 0.82 in the long-term cohort. The classification results of the discriminant analysis were demonstrated by the confusion matrix.

**Conclusions:**

The proposed SVM model indicated that DH, FA, and LLS were statistically associated with a clinical outcome of DLS. These results may provide optimized clinical strategy for treatment of DLS.

## Introduction

As the aging society comes, degenerative lumbar spondylolisthesis (DLS) with symptomatic stenosis presents a serious challenge to healthcare (1, 2). After struggling with the dilemma of decompress treatment, spine surgeons have recognized that it will cause inferior postoperative outcomes without fusion, including persistent pain, pseudarthrosis, and progressive degenerative changes (3–5). Therefore, lumbar interbody fusion (LIF), in combination with decompression, has become the main surgical options for the treatment of DLS.

We noted that the negative feedback from the patients is the main reason for the increased reoperation rate and decreased treatment satisfaction, which result in contradictions or bothers between doctors and patients and heavy substantial burden on the healthcare systems (6).

The current dominant functional scales for evaluating symptoms of degenerative spinal diseases are the Visual Analog Scale (VAS) and the Oswestry Disability Index (ODI) (1, 7–9). However, there are few studies on surgical efficacy evaluation based on patient-reported outcomes (PROs) at present (7).

Machine learning (ML) has proved to be an effective data-driven automatic modeling mechanism in processing numerous problems in the biomedical field (10–13). Support vector machine (SVM) is a well-established classifier, which was applied for accurately patients' classification and treatment options improvement (14).

Considering there is a lack of superior predictive models, the purpose of this study was to investigate the clinical distribution of PROs and to establish a predictive model to evaluate the risk of unfavorable clinical outcomes in short- and long-term postoperative cohorts using the SVM algorithm.

## Methods

### Study Design and Participants

The diagnosis of DLS required a minimum 3-mm vertebral displacement observed in lateral X-rays, and patients were selected from January 1, 2015 to October 1, 2020 at our institution. Inclusion criteria: (1) age ranged from 50 to 80 years old, (2) patients diagnosed with symptomatic DLS, (3) single-level spondylolisthesis and LIF, (4) traceability of clinical parameters, radiological variables, and surgical records, (5) at least 1 year of follow-up available. Exclusion criteria: (1) American Society of Anesthesiologists (ASA) rating of III or higher, (2) multi-level decompression and LIF with pedicle screws, (3) previous lumbar surgical intervention, (4) revision surgery, (5) spinal tumor, deformity, fracture, infection or other spine diseases, and (6) rejection of implants.

The Ethics Committee of our hospital approved this study. All data were extracted from hospital electronic system.

### Data Collection

General patient information was recorded, including age, sex, body mass index (BMI), smoking, diabetes, Oswestry Disability Index (ODI), Visual Analog Scale (VAS), fusion technique, operation level, bone graft methods, cement augmentation, and surgical complications.

**Figure 1:**
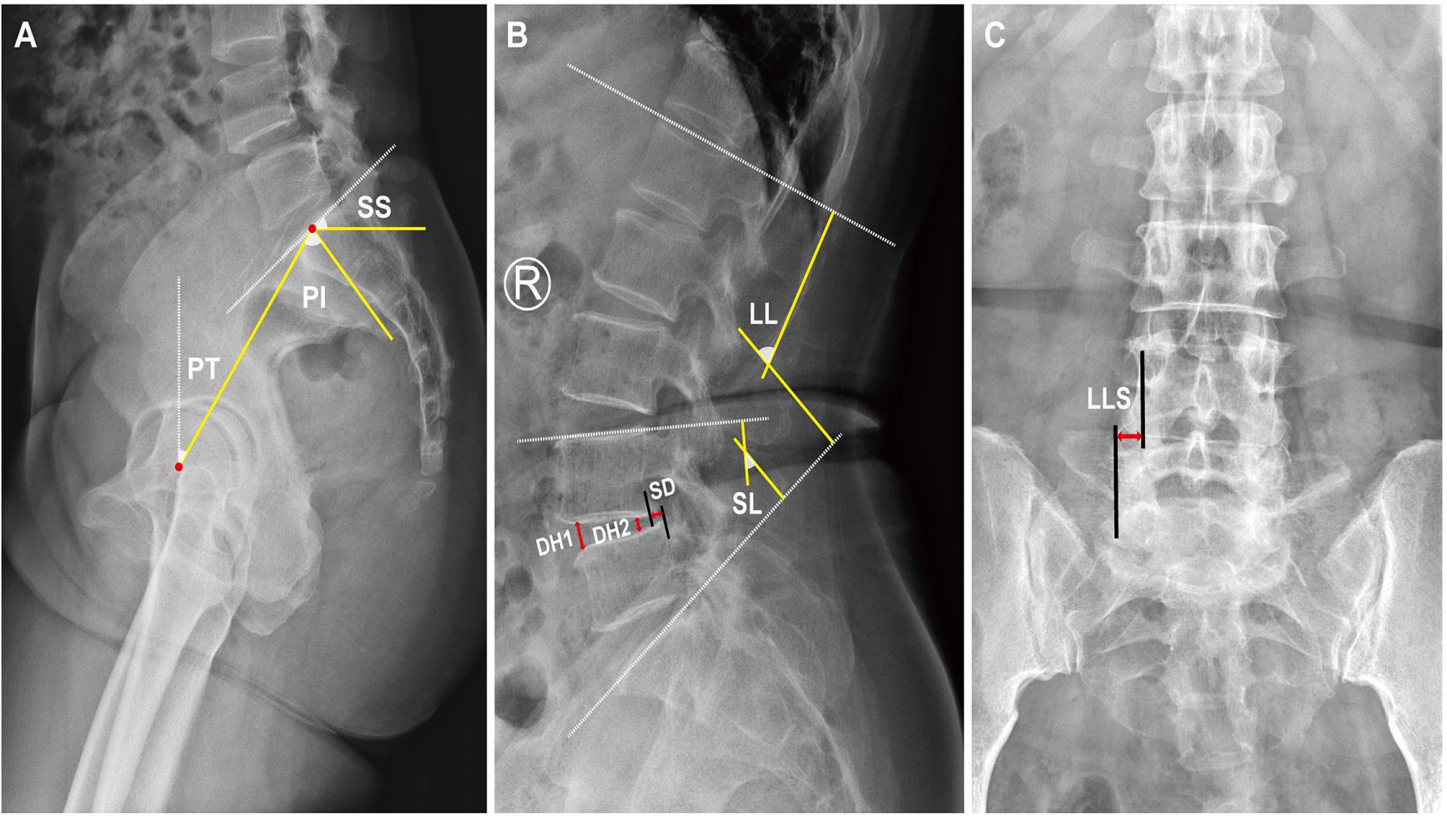
A schematic diagram of spinopelvic parameters, lumbar parameters, and lateral listhesis (LLS). Spinopelvic parameters **(A)** included pelvic incidence (PI), pelvic tilt (PT), and sacral slope (SS). Lumbar parameters **(B)** included lumbar lordosis (LL), segment lordosis (SL), slip degree (SD), and disc height (DH). Lateral listhesis (LLS) was measured according to **(C)**.

**Figure 2:**
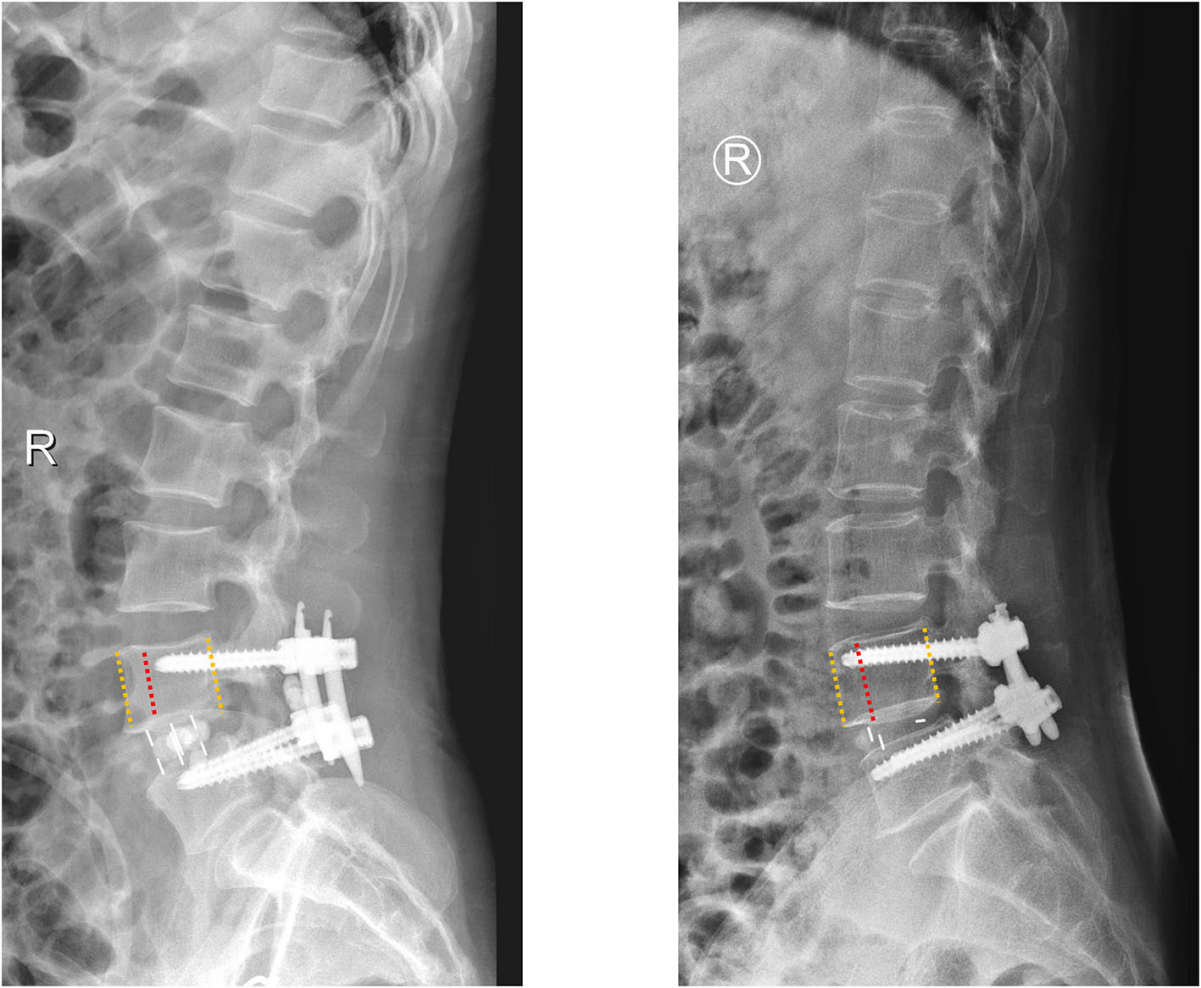
A schematic diagram of insertion depth; the patients were assigned to the S group (the screws accounted for 60–80% of the anteroposterior diameter of vertebral body) and the L group (the screws accounted for more than 80% of the anteroposterior diameter of vertebral body).

**Figure 3:**
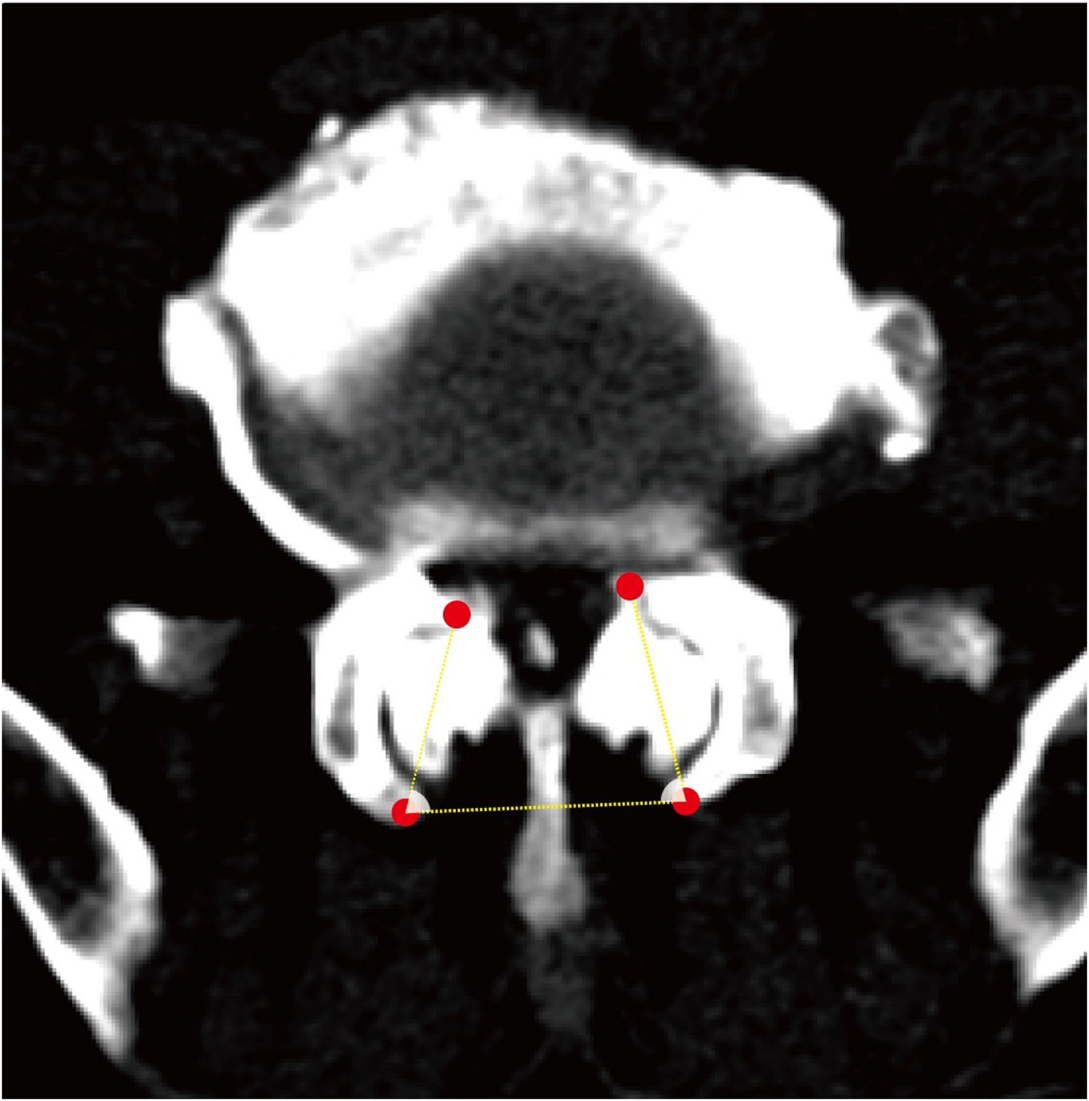
A schematic diagram of the facet angle. A facet line was drawn between the 2 end points of each facet. The angle was measured between the facet line and the line connecting the bilateral dorsal points and recorded the average value.

All patients received a complete imaging evaluation, including standard anteroposterior and lateral X-rays, CT, dual-energy X-ray (DEXR), and MRI. Based on X-rays results, we recorded and analyzed the following radiological variables: sacral slope (SS), the angle between the sacral plate and the horizontal axis. Draw the line connecting the midpoint of the femoral head to the midpoint of the sacral plate, which formed an angle with the vertical line of the sacral plate as pelvic incidence (PI) and an angle with the longitudinal axis as pelvic tilt (PT) (Figure 1A). Slip degree (SD), the horizontal distance of posterior wall between the slipped and upper vertebrae; lumbar lordosis (LL), the angle between the upper endplate of L1 and the upper endplate of S1; segment lordosis (SL), the angle between the upper endplate of the upper vertebrae and the upper endplate of the lower vertebrae of the slipped segment; disc height (DH), the average of anterior disc height and a posterior disc height (Figure 1B); lateral listhesis (LLS) was defined the distance between vertebral bodies (Figure 1C). Insertion depth, as 60% of the vertebral depth, was treated as the minimum screw purchase at our institution; the patients were assigned to the S group and the L group (Figure 2). The facet angle (FA) between the facet line and the line connecting the bilateral dorsal points was measured on CT (Figure 3). Osteoporosis was defined as T-score < −2.5 SD on dual-energy X-ray absorptiometry.

PROs were determined as function evaluations. ODI was used to describe the patient's quality of life; a smaller value suggested better function. VAS was chosen to measure the patient's back pain; a decreased value suggested reduced pain. Based on practical experience and literature review, significant symptomatic improvement was observed in patients with lumbar spondylolisthesis after decompression and fusion. (7) We defined the short-term endpoint event as a 60% improvement in VAS and ODI 2 weeks postoperatively, and long-term endpoint event as a 90% improvement in VAS and ODI 1 year postoperatively.

### Surgical Technique

**Figure 4:**
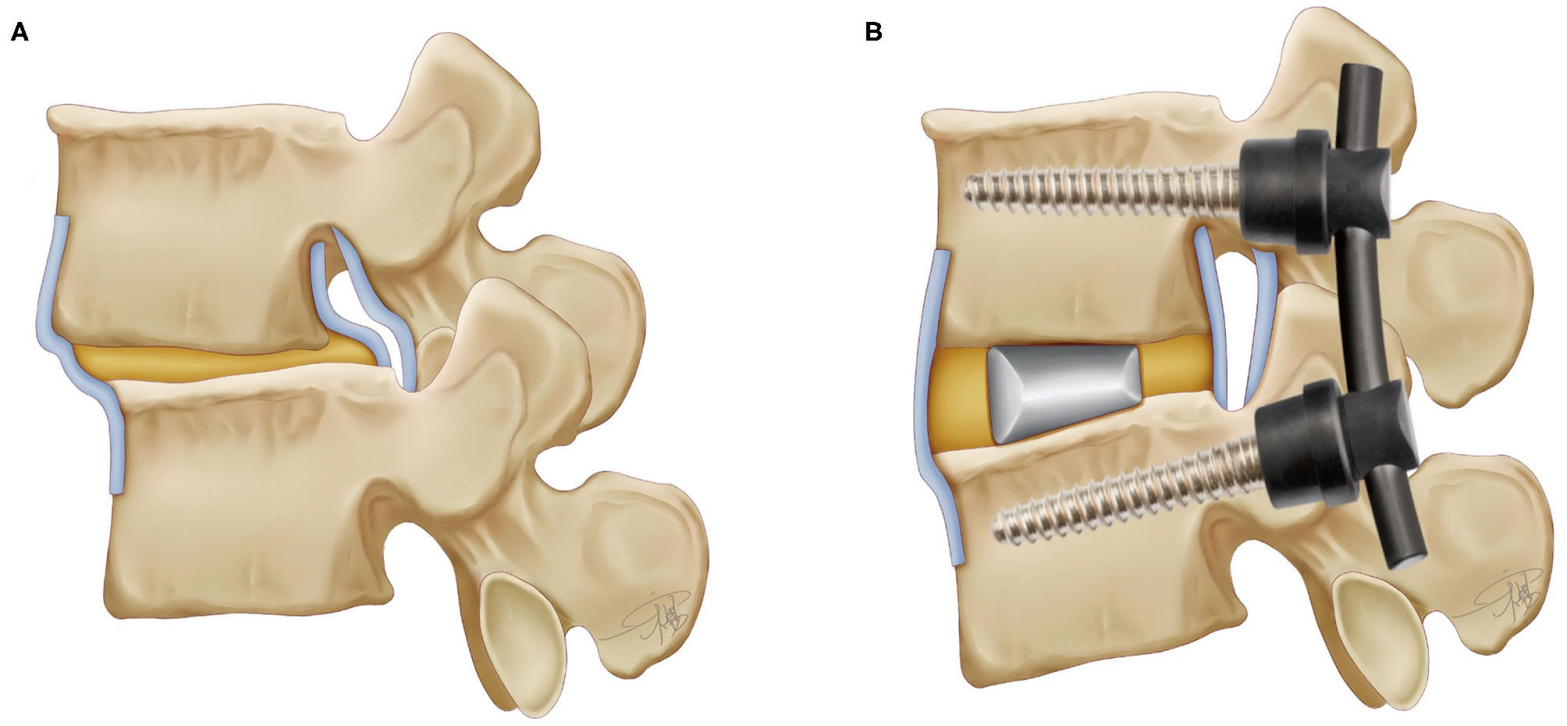
The lumbar spine model and the instrumentations after TLIF technique (the interbody fusion cage and the pedicle screw).

All recruited patients were operated by the same surgeon. Prone position after general anesthesia has taken effect. The transforaminal lumbar interbody fusion (TLIF) group and posterior lumbar interbody fusion (PLIF) group designed their individual landmarks separately with the assistance of C-arm fluoroscopy. The TLIF group used a percutaneous fixation approach to implant screws *via* the pedicle, and PLIF used a conventional open approach. If the primary surgeon felt it necessary (preoperative diagnosis of osteoporosis or intraoperative finding of bone weakness), the tip of the vertebroplasty needle was inserted into the central point of the vertebral body under fluoroscopic guidance, and, approximately, 2–3 ml of polymethylmethacrylate (PMMA) bone cement was injected. The corresponding lamina is removed and retained for bone grafting, and the upper and lower cartilage endplates of the intervertebral disc are scraped with a ring curette after debridement of the annulus fibrosus and nucleus pulposus. The rods are inserted bilaterally, and traction is applied to lift the slipped segment and ensure uniformity with the upper and lower vertebrae as much as possible. Some patients might receive cement augmentation due to screw retraction at this stage. After fluoroscopic demonstration of satisfactory correction of the slippage, an appropriately sized cage is filled with allograft and/or autologous bone, and the processed interbody fusion cage is inserted into the interbody space to support the vertebral body (Figure 4).

### Support Vector Machine

VAPNIK first conceived the SVM technique as a classification method for both linear and non-linear data two decades ago. The input vectors were mapped to a high-dimensional feature space *via* preselected non-linear mappings, and the optimal classification hyperplane was constructed in this space. The support variables eventually obtained are exactly the nearest sample points. The data input format was recorded as “csv” or “xlxs” and analyzed depending on the Python programming language (Python 3.8.0, Python Software Foundation).

### Statistical Analysis

For continuous variables, normal distribution was subject to Student's *T*-tests, and non-normal distribution was subject to Mann–Whitney test. Chi-square test was adopted to analyze categorical variables. Continuous variables and categorical variables are presented as means ± standard deviations (SD) and relative frequencies and percentages, respectively. In addition, we applied logistic regression analysis to identify the predictors of unfavorable clinical outcomes within 2 weeks postoperatively. The statistically significant variables obtained from the univariate regression analysis were again subjected to multivariate regression analysis. Furthermore, the selected variables were put into the SVM classifier. Statistical differences were defined as *p* < 0.05. All statistical analysis was performed with SPSS version 22.0 (IBM SPSS, Armonk, New York).

## Results

From January 2015 to October 2020, a total of 157 consecutive patients met the inclusion criteria after excluding three patients due to follow-up loss. The demonstrative information of the patients was observed in Table 1. The mean age of the patients was 63.84, and the majority was females (approximately two times of males). Based on the selection basis of degenerative disease, 43 patients (27.39%) were diagnosed with osteoporosis, and 52 patients (33.12%) received intraoperative cemented augmentation. All the patients underwent single-segment fusion, with the primary level of fusion at L4/5 and TLIF as the dominant fusion approach. Autograft bone was the preferred bone graft method. Two patients had intraoperative screw retraction, and subsequent short-term poor functional recovery was observed during follow-up. The fusion rate was 95.54%, and, among the seven patients who achieved inadequate fusion, three exhibited consistent low back pain and one underwent revision surgery as the result. According to the PROs, 26 patients and nine patients were eventually assigned to the short- and long-term unfavorable cohorts; the score summary is shown in Table 2. Serious complications, such as neurological deficits, deep vein thrombosis, and wound infection, did not occur in this series of patients. However, we identified two patients with postoperative urinary retention and one patient who underwent revision surgery for persistent low back pain.

**Table 1:** Demonstrative information of the patient cohort.

**Variables**	**Patients (*N* = 157)**
Age (years), mean ± SD	63.84 ± 8.23
Sex (male/female), *n* (%)	52 (33.12%)/ 105 (66.88%)
BMI (kg/m^2^), mean ± SD	24.39 ± 2.32
Smoking, *n* (%)	23 (14.65%)
Diabetes mellitus, *n* (%)	26 (16.56%)
Osteoporosis, *n* (%)	43 (27.39%)
**Fusion level**, ***n*** **(%)**	
L3/4	18 (11.46%)
L4/5	75 (47.77%)
L5/S1	64 (40.77%)
**Fusion approach**, ***n*** **(%)**	
TLIF	82 (52.23%)
PLIF	52 (33.12%)
TLIF + PLIF	23 (14.65%)
**Types of bone graft**	
Autograft bone	78 (49.68%)
Allograft bone	41 (26.12%)
Autograft + allograft bones	38 (24.20%)
Cement augmentation, *n* (%)	52 (33.12%)
Screw retraction, *n* (%)	2 (1.27%)
Fusion rate (fused/unfused), *n* (%)	150 (95.54%)/ 7 (4.46%)
Short-term unfavorable cohort, *n* (%)	26 (16.56%)
Long-term unfavorable cohort, *n* (%)	9 (5.73%)

**Table 2:** Description of changes in DOI and VAS scores.

**Oswestry disability index and the visual analog scale**	**Pre-operation**	**Two weeks after operation (F/UF)**	**One year after operation (F/UF)**
ODI score	63.93 ± 10.35	28.95 ± 10.01 (26.89/ 39.35)	8.17 ± 3.20 (7.89 /12.78)
VAS score	6.96 ± 1.20	2.78 ± 1.26 (2.49/ 4.23)	0.69 ± 0.67 (0.64 /1.44)

*F, favorable clinical outcomes; UF, unfavorable clinical outcomes*.

Table 3 compared the preoperative and postoperative radiological parameters. There were no statistically significant differences both in the short- and long-term cohorts, including SD, LL, and PI. In the present study, we found that DH, LLS, PT, and FA were significantly higher in the long-term unfavorable cohort than in the counterpart, and a higher proportion in the short screw group. In evaluating the long-term clinical efficacy, the differences of DH, LLS, and FA in the unfavorable group were statistically significant.

**Table 3:** Comparison of the radiologic variables.

**Variable**	**Total (*n* = 157)**	**Short-term cohort**			**Long-term cohort**		
		**Favorable group (*n* = 131)**	**Unfavorable group (*n* = 26)**	***p***	**Favorable group (*n* = 148)**	**Unfavorable group (*n* = 9)**	***p***
**DH (mm)**							
Preop	8.60 ± 2.80	8.40 ± 2.80	9.60 ± 2.63	0.045	8.57 ± 2.81	9.00 ± 2.71	0.659
Postop	5.70 ± 1.84	5.22 ± 1.43	8.11 ± 1.75	<0.001	5.57 ± 1.79	7.84 ± 1.14	<0.001
**LLS (mm)**							
Preop	7.67 ± 3.09	6.83 ± 2.01	11.89 ± 4.02	<0.001	7.43 ± 2.91	11.56 ± 3.50	<0.001
Postop	1.98 ± 0.73	1.93 ± 0.63	2.24 ± 1.09	0.172	1.95 ± 0.70	2.53 ± 0.84	0.021
**SD (mm)**							
Preop	17.76 ± 2.85	17.72 ± 2.86	17.94 ± 2.74	0.733	17.77 ± 2.87	17.51 ± 2.37	0.789
Postop	6.86 ± 1.70	6.83 ± 1.72	7.00 ± 1.61	0.629	6.82 ± 1.68	7.40 ± 1.89	0.326
**LL (** **°** **)**							
Preop	38.87 ± 2.25	38.83 ± 2.26	39.10 ± 2.17	0.581	38.92 ± 2.24	38.16 ± 2.27	0.327
Postop	45.26 ± 2.52	45.34 ± 2.51	44.89 ± 2.54	0.413	45.30 ± 2.53	44.62 ± 2.33	0.435
**SL (** **°** **)**							
Preop	16.40 ± 2.79	16.43 ± 2.87	16.24 ± 2.37	0.729	16.38 ± 2.82	16.66 ± 2.26	0.778
Postop	24.64 ±1.65	24.77 ± 1.67	24.01 ± 1.38	0.020	24.67 ± 1.67	24.28 ± 1.13	0.501
**SS (** **°** **)**							
Preop	36.10 ± 2.30	36.20 ± 2.26	35.61 ± 2.42	0.234	36.10 ± 2.29	36.13 ± 2.37	0.973
Postop	37.81 ± 2.83	38.08 ± 2.78	36.48 ± 2.72	0.008	37.87 ± 2.81	36.83 ± 3.04	0.286
**PI (** **°** **)**							
Preop	54.14 ± 2.57	54.31 ± 2.54	53.30 ± 2.56	0.068	54.22 ± 2.58	52.78 ± 1.95	0.104
Postop	52.87 ± 2.39	52.94 ± 2.41	52.49 ± 2.27	0.377	52.91 ± 2.41	52.18 ± 1.94	0.377
**PT (** **°** **)**							
Preop	18.03 ± 2.76	17.97 ± 2.77	18.31 ± 2.69	0.572	18.03 ± 2.75	18.06 ± 3.04	0.977
Postop	15.17 ± 1.44	15.06 ± 1.43	15.78 ± 1.37	0.020	15.14 ± 1.43	15.76 ± 1.49	0.208
Facet angle (°)	57.71 ± 7.29	55.15 ± 6.07	64.59 ± 7.79	<0.001	56.29 ± 6.95	63.65 ± 9.01	0.003
**Insertion depth (%)**				0.010			0.627
S group	103 (65.61%)	80 (61.07%)	23 (88.46%)		96 (64.87%)	7 (77.78%)	
L group	54 (34.39%)	51 (38.93%)	3 (11.54%)		52 (35.13%)	2 (22.22%)	

*Preop, preoperative; Postop, postoperative; DH, disc height; LLS, lateral listhesis; SD, slip degree; LL, lumbar lordosis; SL, segment lordosis; SS, sacral slope; PI, pelvic incidence; PT, pelvic tilt; S group, the screws account for 60–80% of the anteroposterior diameter of vertebral body; L group, the screws accounted for more than 80% of the anteroposterior diameter of vertebral body*.

Univariate and multivariate regression analyses indicated that preoperative LLS (*p* = 0.005), postoperative DH (*p* = 0.004), and FA (*p* = 0.030) were independent risk factors in a short-term adverse outcome (Table 4), while only postoperative DH (*p* = 0.038) was independently associated with a long-term outcome (Table 5).

**Table 4:** Univariate and multivariate logistic regression analysis of unfavorable clinical outcomes in the short-term cohort.

**Variables**	**Univariable logistic**				**Multivariable logistic**			
	**regression analysis**				**regression analysis**			
	**OR**	**95% CI**		***p***	**OR**	**95% CI**		***p***
		**Lower**	**Upper**			**Lower**	**Upper**	
Preop. DH	1.17	1.00	1.36	0.048	0.98	0.67	1.41	0.894
Preop. LLS	2.11	1.51	2.97	<0.001	2.01	1.23	3.28	0.005
Postop. DH	3.28	2.11	5.10	<0.001	2.81	1.40	5.65	0.004
Postop. LLS	1.78	1.00	3.15	0.050	0.87	0.24	3.25	0.855
Postop. SL	0.74	0.56	0.98	0.037	1.01	0.59	1.71	0.979
Postop. SS	0.81	0.69	0.95	0.011	0.72	0.47	1.10	0.133
Postop. PT	1.44	1.05	1.97	0.023	1.23	0.64	2.36	0.544
**Insertion depth**								
S group	Ref	Ref	Ref	Ref	Ref	Ref	Ref	Ref
L group	0.21	0.06	0.72	0.013	0.83	0.08	9.12	0.902
Facet angle	1.23	1.13	1.34	<0.001	1.20	1.02	1.41	0.030

*OR, odds ratio; 95% CI, 95% confidence interval*.

**Table 5:** Univariate and multivariate logistic regression analysis of unfavorable clinical outcomes in the long-term cohort.

**Variables**	**Univariable logistic**				**Multivariable logistic**			
	**regression analysis**				**regression analysis**			
	**OR**	**95% CI**		***p***	**OR**	**95% CI**		***p***
		**Lower**	**Upper**			**Lower**	**Upper**	
Preop. DH	1.06	0.83	1.34	0.657	0.88	0.65	1.20	0.431
Preop. LLS	1.33	1.13	1.57	0.001	1.24	0.98	1.57	0.069
Postop. DH	1.77	1.25	2.50	0.001	1.60	1.02	2.49	0.038
Postop. LLS	2.80	1.13	6.98	0.027	1.43	0.55	3.69	0.465
Postop. SL	0.86	0.56	1.32	0.864	1.14	0.67	1.94	0.619
Postop. SS	0.88	0.68	1.12	0.289	0.91	0.67	1.24	0.566
Postop. PT	1.37	0.84	2.24	0.214	1.03	0.58	1.82	0.919
**Insertion depth**								
S group	Ref	Ref	Ref	Ref	Ref	Ref	Ref	Ref
L group	0.53	0.11	2.63	0.435	1.58	0.23	10.93	0.645
Facet angle	1.14	1.04	1.25	0.006	1.03	0.92	1.16	0.557

*OR, odds ratio; 95% CI, 95% confidence interval*.

**Figure 5:**
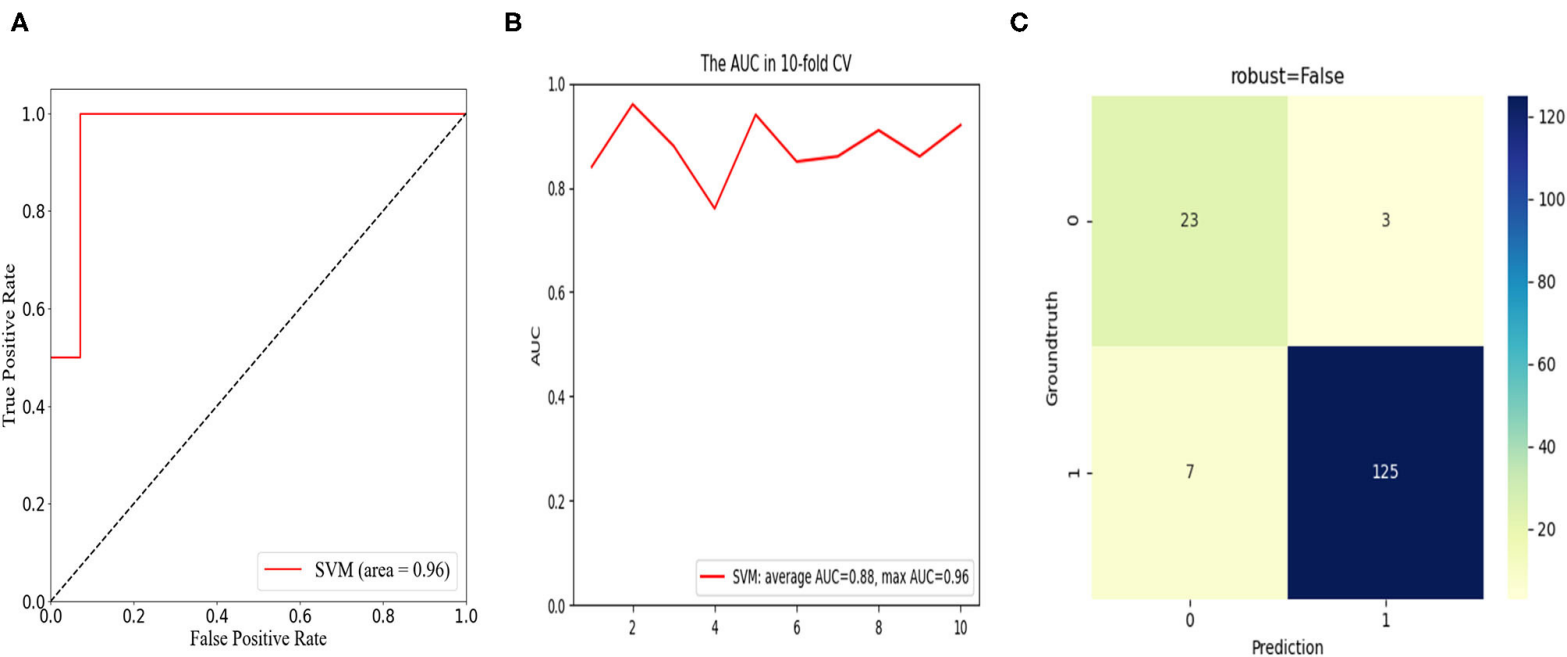
In the short-term cohort, receiver operation characteristic (ROC) curve analysis of the SVM model with the maximum value of 0.96 **(A)**; ROC curve analysis of 10-fold cross validation of the SVM model for predicting the risk of unfavorable clinical outcomes following LIF with average AUC of 0.88 and max AUC of 0.96 **(B)**; confusion matrix of the SVM model **(C)**.

**Figure 6:**
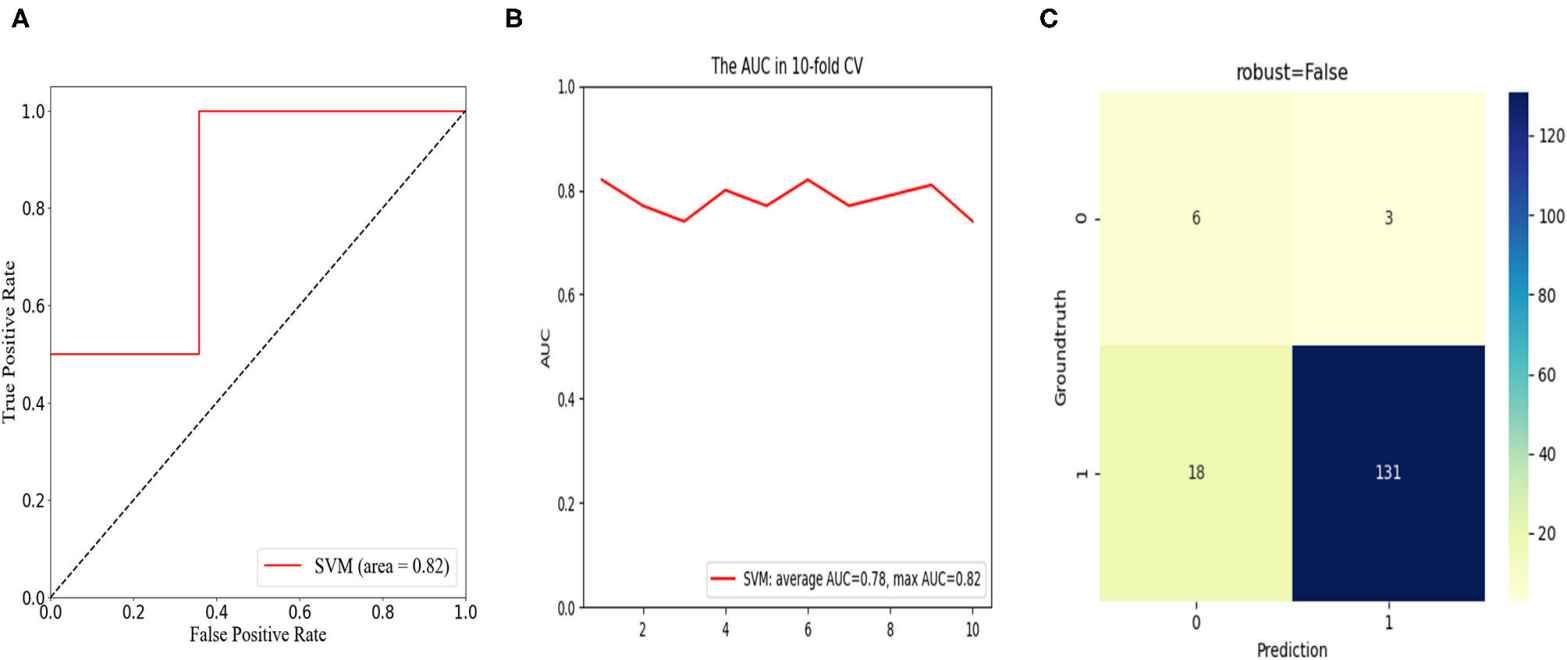
In the long-term cohort, receiver operation characteristic (ROC) curve analysis of the SVM model with the maximum value of 0.82 **(A)**; ROC curve analysis of 10-fold cross validation of the SVM model for predicting the risk of unfavorable clinical outcomes, following LIF with average AUC of 0.78 and max AUC of 0.82 **(B)**; confusion matrix of the SVM model **(C)**.

To predict the risk of an adverse outcome in patients with spondylolisthesis, we developed SVM models based on the above-mentioned independent predictors. We evaluated the ROC curve of the model and calculated the area under the ROC curve. Furthermore, 10-fold cross-validation was performed to evaluate the predictive power of the algorithm. For patients who fed back adverse PROs in the short term, the SVM model showed satisfactory classification performance with mean and maximum AUC values of 0.88 and 0.96, respectively (Figure 5). Among all routine indicators, the only predictor of a long-term outcome (1 year) was postoperative DH, and the long-term SVM model showed slight weakening of performance compared to the short-term SVM, with mean and maximum AUC values of 0.78 and 0.82, respectively (Figure 6). Based on the confusion matrix, these indices provide evidence for performance evaluation, and the following equations are presented:


$$\begin{array}{l} Accuracy\;\left(ACC\right)=\frac{TP+TN}{TP+TN+FP+FN}\\ \;Precision=\frac{TP}{TP+FP} \end{array}$$

The confusion matrix showed the models were excellent at predicting the risk of unfavorable clinical outcomes in patients with spondylolisthesis with the ACC of 93.7% and precision of 88.5% in the short-term cohort and the ACC of 86.7% and precision of 66.7% in the long-term cohort.

## Discussion

No consensus has been achieved on whether to perform interbody fusion for degenerative lumbar spondylolisthesis. Chan et al. (15) concluded that their evidence that supported decompression alone was a more beneficial intervention to minimize surgery-related trauma and postoperative morbidity, and had comparable efficacy to additional interbody fusion, especially for low-grade spondylolisthesis. Ghogawala et al. controverted this conclusion (5). After 4-year follow-up, they found decompression with lumbar fusion was associated with a significant improvement in physical health-related quality of life compared to laminectomy alone. The abnormal sagittal alignment of the lumbar spine can lead to increased energy demands in non-resting positions, resulting in early fatigue and exercise intolerance (9). Therefore, the aim of DLS surgery is to restore the normal anatomical sequence and stability of the lumbar spine based on the removal of spinal stenosis (7). In the present study, all the patients underwent decompressive laminectomy and instrumented lumbar spinal fusion to achieve the above-mentioned treatment goals. PROs were direct and convenient evidence to measure the quality of DLS surgeries. Meanwhile, the VAS and ODI scores were regarded as the primary evaluation tools; these subjective data offered “real world” insights into guiding and improving surgical techniques (15, 16). Moreover, a series of reported model evaluation metrics indicate that the deep learning can provide satisfactory performance in distinguishing target patients, demonstrating good clinical application (17, 18). A broad application prospect requires additional data science techniques to support, including supervised dimensionality reduction for big data (19), deep learning networks for diabetic retinopathy (20), and identification of malnourished patients (21). Using the SVM model, postoperative DH, FA, and preoperative LLS were eventually identified as risk factors in unfavorable clinical outcomes at short-term follow-up, whereas only postoperative DH predicted clinical efficacy at long-term follow-up.

A large number of studies have demonstrated the importance of maintaining DH for achieving vertebral stability either in decompression alone or decompression with interbody fusion (3, 9). Our study indicated that the patients that attained a <6-mm DH intraoperatively were more likely to derive a sustained benefit from the decompression with a fusion procedure than the patients reporting poor clinical outcomes. The mean DH was higher in the short-term adverse group (8.11 vs. 7.84), which could be acceptably explained by progressive degeneration at the operated segment. The definition of the pathological change is adjacent segment disease (ASD), which was the condition responsible for half of the reoperations (3, 22, 23). Although PROs suggested that ASD was an unavoidable healthcare concern, we still feel encouraged by a reoperation rate of <1%. Sato et al. reported a reoperation rate of 2.2% in their 1-year follow-up (3), while this incidence reached a staggering 10% in the study by Deyo et al. (24). Furthermore, classifying patients with > 60% improvement in functional evaluation as the definitive efficacy group at 2 weeks postoperatively, we considered 6 mm as an effective threshold associated with clinical outcomes, which was consistent with previous studies (3, 25, 26). Considering the intervertebral space could serve to guide the selection of an interbody fusion cage, a careful fluoroscopic image review after restoring the slipped vertebra is necessary. Interestingly, Yen et al. reported the intradiscal vacuum phenomenon (IVP) occurring after interbody fusion was associated with increased disc height and vertebral instability (27). An intensive biomechanical analysis of this susceptible anatomy seems imperative.

The structure, locating on the posterolateral region of the vertebral body and connecting the adjacent vertebral arch, is the facet joint, which constitutes the classic triple-joint complex with the intervertebral disc (28). In the current study, outcomes in consecutive patients suggested that increased FA limits functional recovery in the short term without impacting on long-term outcomes. It was worth noting that 5–90% of chronic low back pain was triggered by facet joint pain (29). For symptomatic DLS, lumbar degenerative changes and facet joint misalignments are mutually anatomically predisposing factors (30). Thus, the facet joints, which bear approximately a quarter of the relevant axial load, play an important but neglected role in reflecting the PROs (29), and this was particularly evident in the case of short-term assessments. This variable conceals its function when focusing on and attempting to improve long-term PROs. Assuming FA does not actually worsen surgical outcomes (31), a more convincing association was acknowledged. Due to the local fixation with pedicle screws, the altered facet joint stress accelerated disc degeneration, leading to an additional influence of disc height in predicting long-term outcomes (28). This explanation was also corroborated in this study. The finite element analysis from Park et al. externally verified the homogeneity between DH and FA (32). Moreover, in the current healthcare system, pain management has become an important part of surgical intervention, especially in the treatment of degenerative spinal diseases, and has shown promising results (28, 33–35). Nevertheless, for postoperative patients, pain is a generalized clinical symptom. It is necessary for the spine surgeons to cautiously analyze the etiology of the patient's adverse PROs to achieve precise management.

In general, successful surgery is largely dependent on acquiring a solid fusion and re-establishing normal local sagittal and coronal balance. The short-term postoperative outcome was correlated with preoperative LLS but independent of postoperative LLS. In other words, the difference between the two groups postoperatively was not statistically significant (1.93 vs. 2.24, *p* = 0.172), and measurement of preoperative coronal imbalance was more valuable. The role of sagittal lumbar alignment in assessing disease grade and deciding on individualized surgical strategies was well recognized in published articles (1–3, 7), but coronal malalignment was an equally important issue to be treated with caution (1, 36, 37). Although we identified the presence of LLS preoperatively and targeted the restoration of normal lumbar coronal balance by obtaining greater lumbar lordosis intraoperatively (38), the consideration was reflected in the comparison of postoperative data, but an inferior PRO was still observed. There were several conceivable reasons for the poor outcomes. First, asymmetric degenerative changes lead to disc collapse and laxity of the paravertebral ligaments, manifested by LLS of vertebral body and lateral displacement of the disc (1). This complicated deformity had more severe pathological changes than a mere sagittal imbalance. A limited surgical benefit was observed in DLS with LLS, including an elevated and persistent back pain due to the coronal displacement of the slipped segment (36). Second, purposeful surgical strategies and instruments were recommended to correct vertebral slippage in patients with lateral slippage, including preserving the lumbar lordosis, implanting a larger cage and laterally distracting the displaced vertebral body (1, 39). However, higher restoration requirements were frequently associated with increased risk of major complications, including extensive soft tissue injury and intraoperative blood loss, all of which can reduce short-term PROs (9). Therefore, alleviating the symptoms of DLS with LLS through a modified surgical approach remained a challenge that required an all-out effort to address.

Combining SVM machine learning technique to analyze predictors of short- and long-term adverse clinical outcomes from routinely available variables was the innovation of this study. Ten-fold cross-validation AUC and confusion matrix suggested that our model performed well. However, there were several limitations in our study. First, selection bias may exist due to the inherent flaw of retrospective studies. Second, the analyzed data were from our single institution, resulting in a lack of adequate training of the SVM model. Therefore, future studies may require a multicenter prospective randomized controlled design to assist in externally validating the credibility of our conclusion. Finally, despite an attempt to analyze extensive patient information, spinal degeneration is a multidimensional and progressive disease that still left some data unaccounted for.

## Conclusion

In conclusion, both TLIF and PLIF are practicable surgical strategies to improve symptoms in patients with DLS. Postoperative DH, FA, and preoperative LLS were statistically associated with the short-term clinical outcome, while only postoperative DH accurately predicted the long-term clinical outcome with an average follow-up time of 1 year. The proposed SVM model showed superior predictive performance.

## Data Availability Statement

The raw data supporting the conclusions of this article will be made available by the authors, without undue reservation.

## Ethics Statement

The study was approved by an Institutional Ethics Committee at the First Affiliated Hospital of Dalian Medical University. Considering that this work was a retrospective study, the Ethics Committee waived the requirement for informed consent from patients.

## Author Contributions

SD and YZ completed the study design. SD, YZ, HY, and NT performed the study and collected and analyzed the data. SD, YZ, and GH drafted the manuscript. JL and KT provided the expert consultations and suggestions and conceived the study, participated in its design and coordination, and helped to embellish language. All the authors reviewed the final version of the manuscript.

## Funding

This work is supported by National Natural Science Foundation of China (No. 81601901) and Natural Science Foundation of Liaoning (No. 2019-MS-079 and No. 20170540285).

## Conflict of Interest

The authors declare that the research was conducted in the absence of any commercial or financial relationships that could be construed as a potential conflict of interest.

## Publisher's Note

All claims expressed in this article are solely those of the authors and do not necessarily represent those of their affiliated organizations, or those of the publisher, the editors and the reviewers. Any product that may be evaluated in this article, or claim that may be made by its manufacturer, is not guaranteed or endorsed by the publisher.

## Abbreviations

DLS: Degenerative lumbar spondylolisthesis
LIF: Lumbar interbody fusion
SVM: Support vector machine
ASA: American Society of Anesthesiologists
BMI: Body mass index
LLS: Lateral listhesis
ODI: Oswestry Disability Index
VAS: Visual Analog Scale
PROs: Patient-reported outcomes
CT: Computed tomography
DEXR: Dual-energy X-ray
MRI: Magnetic resonance imaging
SD: Slip degree
LL: Lumbar lordosis
SL: Segment lordosis
SS: Sacral slope
PI: Pelvic incidence
PT: Pelvic tilt
DH: Disc height
FA: Facet angle
TLIF: Transforaminal lumbar interbody fusion
PLIF: Posterior lumbar interbody fusion
PMMA: Polymethylmethacrylate.
